# Extra-Uterine Pregnancy

**Published:** 1829-01

**Authors:** 


### EXTRA-UTERINE PREGNANCY.

E. Haydn, aged twenty, the mother of one child, now eighteen months old, was admitted, October 29th, into Guy's Hospital, under the care of Dr. Bright.

Her illness had been of six months' continuance, but had not been very severe until within the last three weeks.

From her admission to the period of her death, she laboured under symptoms which, while they were principally referrible to some disorganization of the thoracic and abdominal viscera; were still indistinct. The most constant symptom was the frequent evacuation of a fluid of a dark grumous appearance. She had also dyspnœa, and occasional tenderness of the abdomen; but of the latter no trace was remaining a few days previous to her death.

The treatment, which was directed principally to the intestinal affection, is uninteresting: it appeared to exercise no control over the symptoms.

Death took place in the night of November 15th, and the following morning, while the body still retained some warmth, it was examined by Dr. Hodgkin.

In the abdomen, a large portion of the peritoneum lining the parietes presented a very dark appearance, less intense superiorly, but deepening towards the pubic region. Numerous adhesions were found, evidently varying in the periods of their formation: these were fewer in the umbilical region, firmer and more numerous towards the pubes. In the left side of the lower belly, these adhesions were so numerous and extensive as to form a perfectly shut cavity, bounded by the sigmoid flexure of the colon, the rectum, the bladder, the lateral and anterior parietes of the abdomen and pelvis, in their respective situations. This cavity enclosed a fœtus, tolerably well formed, of about three months' growth, attached, by an umbilical cord of natural length, to a mass of the size of an egg, apparently performing the office of placenta. These parts, like the cavity itself, were all of a dark brown colour, apparently from a process of decomposition, and the parietes of the cavity were loose and soft, so as readily to separate into shreds.

This cavity communicated with the intestinal canal by two openings, of which the smaller entered the rectum; while the larger, which was two or three inches in length, was in the sigmoid flexure of the colon.

The mucous lining of these intestines was healthy, except at the very margin of the apertures. The uterus was healthy, and afforded no trace of tunica decidua; it was not adherent to the rectum. Nothing remarkable was observed in the ovaries or fallopian tubes, except that, attached to one of the latter, there was a thin membranous cyst, ruptured and collapsed, which had probably contained the fœtus from its formation until it became free in the abdomen, and was enclosed in the new cavity in which it was found.

